# Does Oxygen Feature Chalcogen Bonding?

**DOI:** 10.3390/molecules24173166

**Published:** 2019-08-30

**Authors:** Pradeep R. Varadwaj

**Affiliations:** 1 Department of Chemical System Engineering, School of Engineering, The University of Tokyo 7-3-1, Tokyo 113-8656, Japan; prv.aist@gmail.com or; 2 The National Institute of Advanced Industrial Science and Technology (AIST), Tsukuba 305-8560, Japan

**Keywords:** oxygen-centered chalcogen bonding, sigma-hole intermolecular interactions, first-principles study, QTAIM, NBO, and RDG analyses, bonding characterizations

## Abstract

Using the second-order Møller–Plesset perturbation theory (MP2), together with Dunning’s all-electron correlation consistent basis set aug-cc-pVTZ, we show that the covalently bound oxygen atom present in a series of 21 prototypical monomer molecules examined does conceive a positive (or a negative) σ-hole. A σ-hole, in general, is an electron density-deficient region on a bound atom M along the outer extension of the R–M covalent bond, where R is the reminder part of the molecule, and M is the main group atom covalently bonded to R. We have also examined some exemplar 1:1 binary complexes that are formed between five randomly chosen monomers of the above series and the nitrogen- and oxygen-containing Lewis bases in N_2_, PN, NH_3_, and OH_2_. We show that the O-centered positive σ-hole in the selected monomers has the ability to form the chalcogen bonding interaction, and this is when the σ-hole on O is placed in the close proximity of the negative site in the partner molecule. Although the interaction energy and the various other 12 characteristics revealed from this study indicate the presence of any weakly bound interaction between the monomers in the six complexes, our result is strongly inconsistent with the general view that oxygen does not form a chalcogen-bonded interaction.

## 1. Introduction 

Oxygen is a biologically relevant species [1,2,3,4] that is often negative in molecules. Since it is the first element of the chalcogen family, its electronegativity is very high (3.44) [5]. The reactive profile of the atom has been extensively studied in the field of noncovalent interactions [6,7,8,9,10], especially as a Lewis base (viz. H_2_O) [11].

Since oxygen promotes the making of chemical interactions, it has caused the emergence of versatile chemical systems of supramolecular [12,13], polymeric [14,15], and biological origins [16,17,18]. It also plays a vital role in proton transfer reactions [19,20]. Without a doubt, it serves as a Lewis base for a Lewis acid for the promotion of an intermolecular interaction. For instance, both the α-helix and β-sheet are formed through the O⋯H hydrogen bonding between the amide hydrogen and the amide carbonyl oxygen [21,22,23,24]. Similar topologies of hydrogen bonds have been responsible for the development of various research fields such as crystal engineering [6], molecular recognition [7] and catalysis [8]. For example, the anions in the crystal structures of organic hydrogen l-malate salts, [AH][Hmal] (where A = (4-methyl)benzylamine, (4-chloro)benzylamine, and (3-chloro)benzylamine) create extended architectures (infinite layers) via the O–H⋯O hydrogen-bonded interactions [25]. A question arises as to whether there is any chemically relevant opportunity for the oxygen atom in molecules to behave as a Lewis acid, and whether such a reactive profile allows it to attract the negative Lewis base(s), leading to the formation of the chalcogen bonding interaction [26,27,28].

A chalcogen bond is formed when the positive site on the covalently bound chalcogen atom Ch in the R-Ch molecule is engaged attractively with the negative site in the other molecule, where R is the remainder part of the molecule [26,27,28]. This general definition (i.e., the attraction between sites of opposite polarity) is analogous to that of hydrogen bonds [29], halogen bonds [30] or pnictogen and tetrel bonds [31]. In the latter, the positive site on the covalently bound hydrogen atom, the halogen atom, or the tetrel atom makes an attractive engagement with the Lewis base (viz. N_2_ and NH_3_).

The topology of a chalcogen bond is a subclass of the so-called σ-hole interactions [32,33]. A σ-hole is an electron density-deficient region that appears on the surface of an atom opposite to the extension of the R–M σ-bond, where M is any main group element, for example [33]. A number of studies have conducted in the past, in which the chalcogen bonds were demonstrated originating from the σ-holes localized only on the electron-deficient sulfur, selenium, and tellurium atoms of the Group 16 in molecules [34,35,36,37,38]. However, it has persistently been claimed in several such studies that the first element of this group, i.e., the oxygen in molecules, does not form such a bond, as it does not feature a positive σ-hole [34,35,36,37,38]. To us, this widespread misconception that oxygen does not form chalcogen bonding has already narrowed the scope of theoretical attempts to study oxygen-centered chalcogen bonds in molecular complex systems. The author recognizes that there is almost a very few studies viable in the noncovalent chemistry literature that reported the characteristic features of O-centered chalcogen bonding interactions in chemical complex systems.

This paper attempts to show theoretically using first-principles calculations that whereas the O atom in widely recognized molecules is often observed to be negative, as in OH_2_, this should not be taken as granted for all occasions. We illustrate this by examining a set of 21 chemically important molecules that comprise the O atom. In particular, we show that the O atom in these systems conceives either a negative or a positive region, or both, which appears around the periphery or along the outer extension of the R–O covalent bond. The positive site on the outer extension of the covalently bound O atom has the ability to temper attraction when placed in the close vicinity of the negative site in the partner molecule, thereby causing the formation O-centered chalcogen bonding interaction. We demonstrate this by examining the geometrical, electronic, energetic, vibrational, orbital and charge density topological properties of six binary complexes, which were formed between some of the randomly chosen monomers of the above 21-monomer series and the widely known Lewis bases such as N_2_, NH_3_, PN, and OH_2_.

## 2. Computational Details

The second-order Møller–Plesset perturbation theory (MP2) [39], together with Dunning’s all-electron correlation basis set, aug-cc-pVTZ, was used to energy-minimize the geometries of the 21 monomers (Text T1 of Supplementary Information summarizes the detail of the optimized geometries).

Six binary complexes were also energy minimized at the same level of theory, which were formed between five randomly chosen monomers of this series above and the nitrogen or oxygen bases in N_2_, NH_3_, PN, and OH_2_ (Text T2 of Supplementary Information summarizes the detail of the optimized geometries). We did so in order to determine if the positive site on O has the ability to form a complex with the negative site on these four bases. The Hessian second-derivative calculation was performed for all the monomer and complex systems to examine the nature of their optimized geometries.

The Molecular Electrostatic Surface Potential (MESP) [40] model was adopted to explore the local nature of electrostatic surface potential, and hence to provide insight into the nature of the reactive sites on the monomer molecules. Traditionally, the positive and negative signs of the local minimum and maximum of the electrostatic potential (*V_S,min_* and *V_S,max_*, respectively) have been invoked to characterize the positive and negative sites on the molecular surfaces, respectively [33,41,42,43,44,45]. Note that the sign and magnitude of potential on the electrostatic surface of a main group atom M along the outer portion of the R–M bond extension is generally used to characterize the nature and quantify the strength and size of its σ-hole, respectively [42,43,44,45,46,47]. The larger the *V_S,max_* associated with a site on M, the stronger its interaction with the Lewis base might be [43,46,47]. Whereas the choice of an isodensity envelope is arbitrary [33,48], we used the 0.001 au isodensity envelope on which to compute the electrostatic potential. The Multiwfun software [49] was used to calculate the maxima and minima values of the potential and the AIMAll software [50] was used for the graphical generation of MESPs. The same wavefunction file for each monomer system generated using Gauassian 09 [51] was supplied to both Multiwfun and AIMAll. The Gaussian 09 [51] optimized MP2 geometries of all the 21 monomers were utilized.

Quantum Theory of Atoms in Molecules (QTAIM) [52] and Reduced Density Gradient (RDG) [53] calculations were performed at the same level of theory to evaluate the charge density and isosurface topologies of bonding interactions in the complexes, respectively. It has already been prioritized in several occasions that both the approaches provide reasonable bonding scenarios in complex systems [33,45]. The bond path topology predicted by QTAIM may be missed between bonded atomic basins that are weakly bound with each other, whereas we show in the following section that this is not always the case for the complex systems studied. The molecular graph and charge density characteristics were evaluated using AIMAll software [50], while the RDG isosurface topology of bonding interactions was analyzed using Multifun [49] and VMD [54] software.

The formation of a binary complex involves orbital interaction between a Lewis base and a Lewis acid. To this end, an analysis of the results that emerged from the second-order perturbation theory of Fock Matrix on an NBO basis was carried out. We did so in order to examine the charge transfer delocalization (stabilization) energies *E*^2^ between “filled” (donor) Lewis-type NBOs and “empty” (acceptor) non-Lewis type NBOs in the six binary complexes. Equation (1) describes *E*^2^, where *q_i_* is the donor orbital occupancy, *ε_i_* and *ε_j_* are diagonal elements (orbital energies) associated with each donor NBO (*i*) and acceptor NBO (*j*), respectively, and *F*(*i*,*j*) is the off-diagonal NBO Fock matrix element. The *E*^2^ were computed at the restricted Hartree–Fock (RHF) level with Gaussian 09’s NBO Version 3.1 [55].

(1) $$E^{2}=\Delta E_{ij}=q_{i}\frac{F{\left(i,j\right)}^{2}}{\varepsilon_{i}-\varepsilon_{j}^{}}$$

The density functional theory (DFT) based symmetry-adapted perturbation theory (SAPT) energy decomposition analysis (EDA) [56,57] was carried out to uncover the importance of specific decomposed energy components contributing to the interaction energies of the six binary complexes examined. This approach dissects the interaction energy Δ*E* (SAPT0) of a complex into four major components: electrostatic (*E*_eles_), exchange (*E*_exch_), polarization/induction (*E*_pol_), and dispersion (*E*_disp_), and is approximated by Equation (2). The PSI4 code [57] was used

Δ*E* (SAPT0) = *E*_eles_ + *E*_exch_ + *E*_pol_ + *E*_disp_ (2)

## 3. Results and Discussion

**Figure 1:**
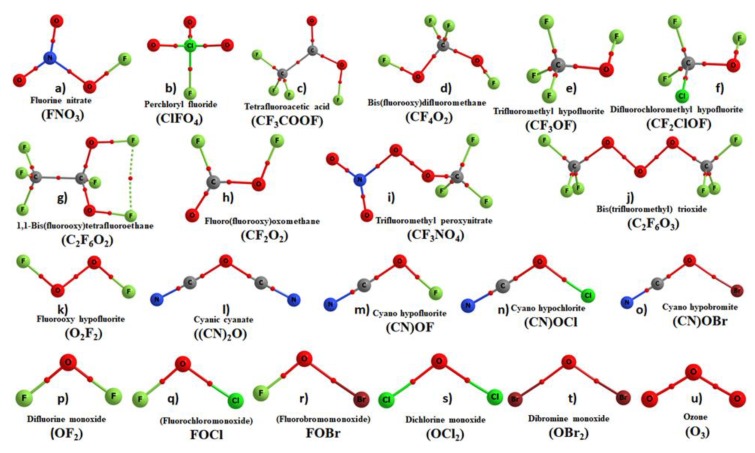
QTAIM molecular graphs of all the 21 monomer molecules examined, obtained on their corresponding MP2/aug-cc-pVTZ optimized geometries. Atom labeling is shown. The solid and dotted lines in atom color represent the bond paths, and the tiny red spheres between atomic basins represent the bond critical points.

The QTAIM molecular graphs of the series of 21 molecules considered in this study are illustrated in Figure 1, showing the presence of covalent links between bonded atomic basins. These links are realized by the presence of the bond path and bond critical point (bcps) topologies of charge density, as well as of significant charge density localization at the bcps. Each of these molecules comprise one, two, or three O atoms. Each has its own significance in the area of synthetic organic or inorganic chemistry. For instance, fluorine nitrate (FONO_2_) was synthesized by Cady [58] and theoretically explored by others [59]. The compounds such as perchloryl fluoride (FClO_3_, b) [60], bis(fluoroxy)perfluoromethane (CF_4_O_2_, d) [61], difluorochloromethyl hypofluorite (CF_2_ClOF, f) [62], trifluoromethyl peroxynitrate (CF_4_NO_4_, i) [63], fluorooxy hypofluorite (O_2_F_2_, k) [64], and others have analogous chemical significance [65]. Trifluoroacetic acid (CF_3_OF, e) is the anionic ion-pairing reagent of choice for peptide separations by reversed-phase high-performance liquid chromatography [66]. Similarly, ozone (O_3_, u) is an important atmospheric molecule that is of crucial importance for Earth’s climate, but also as a UV filter protecting everything living on the Earth, and its reactivity with other systems has been analyzed numerously [67,68,69].

**Figure 2:**
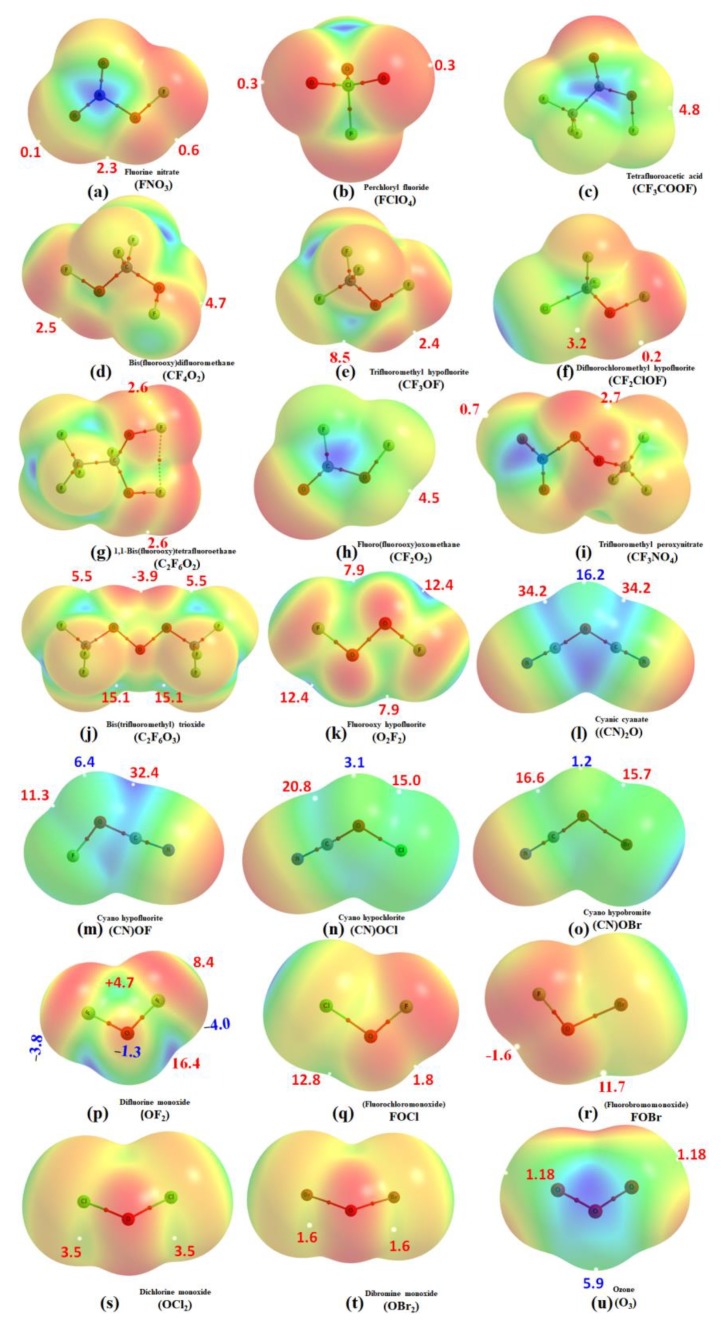
MP2/aug-cc-pVTZ computed 0.001 a.u. isodensity envelope mapped potential on the molecular electrostatic surfaces of 21 monomers. Selected *V_S,max_* (red) and *V_S,max_* (blue) values are shown in kcal mol^−1^, marked by tiny white dots.

Nevertheless, Figure 2 shows the MESPs of all the 21 monomers. The σ-hole on the oxygen is evident in all of them. In some cases, it is near either neutral or absent, and in other cases, it is either negative or positive (moderately weak or strong). This view is emerged from the sign and magnitude of *V_S,max_* being the measure of the nature and strength of the σ-hole, respectively [32,33,41,42,43,44]. As such, the σ-hole on the O atom is near neutral or absent on the N–O bond extensions of the –NO_2_ fragment in FNO_3_ (a). The neutral nature of the σ-hole may be understood based on the argument that the electronegativity and electron-withdrawing properties of the N and O atoms are comparable.

Similarly, the σ-hole is negative on the O–O and Br–O bond extensions in C_2_F_6_O_3_ (j) and BrOF (r), respectively. It is positive and weak on the (C)F–O bond extensions in CF_3_OF (e), CF_2_ClOF (f), and in several others (viz. OCl_2_ (s), OBr_2_ (t), and O_3_ (u)). For instance, the *V_S,max_* associated with the σ-hole on the Cl–O and C–O extensions in FClO_4_ (b) and CF_2_ClOF (f) are +0.3 and +0.2 kcal mol^−1^, respectively, which are indeed positive and weak.

Several molecules of the 21-monomer series comprise σ-holes on the O atom that are very strong. For example, the σ-holes lying along the outer portions of the O–O bond extensions in O_2_F_2_ (k) and C_2_F_6_O_3_ (j) are very large and positive (*V_S,max_* 12.4 and 15.1 kcal mol^−1^, respectively). Their strengths may be comparable with those observed on the C–O bond extensions in (CN)_2_–O (l), F(CN)–O (n), Cl(CN)–O (o), and Br(CN)–O (p), as well as those observed on the X–O (X = F, Cl, Br) bond extensions in (CN)F–O (m), (CN)Cl–O (n), (CN)Br–O (o), ClF–O (q), and BrF–O (r). The strongest σ-hole is observed on the (CN)_2_–O bond extensions (*V_S,max_* = +34.2 kcal mol^−1^, l), which is obviously because that the –CN group in this molecule is highly electron-withdrawing.

The oxygen in the halogen (–X) and cyanide (–CN) substituted OH_2_ monomers in l) to o) is entirely positive. This means both the lateral and axial sites of the O atom in these molecules are positive. The positive *V_S,min_* associated with the lateral portion of the O atom decreases in this order: (CN)_2_–O (+16.2 kcal mol^−1^) > F(CN)–O (+6.4 kcal mol^−1^) > Cl(CN)–O (+3.1 kcal mol^−1^) > Br(CN)–O (+1.2 kcal mol^−1^). The ordering suggests that as the size, and hence the polarizability, of the halogen –X (F, Cl, Br) increases in the series from F through Cl to Br, which replaces the –CN fragment in (CN)_2_–O, the *V_S,min_* associated with the lateral portions of the O decreases in the same order. This is consistent with the trend in the electron-withdrawing ability of the halogen: (–CN >) –F > –Cl > –Br. On the other hand, the *V_S,max_* associated with the axial site of the same O atom in these four molecules are positive. These results show that the development of the nature and strength of the σ-hole is tuned based on the combined effects of the electron-withdrawing, electronegativity, and polarizability of the atoms bound to the O atoms in these molecules. Since both *V_S,max_* and *V_S,min_* are positive and their magnitudes are dissimilar on the O atom, one might conclude that the surface of the O atom in these molecules is accompanied by an anisotropy in the distribution of the charge density [70].

Similarly, and as found for (CN)_2_–O (l), the middle O atom in the O_3_ molecule in (u) is entirely positive. The lateral portion of this atom is significantly more positive than the axial counterparts. The latter ones (axial) do not comprise any σ-hole, whereas the former (lateral) is accompanied by the *V_s,min_* of +5.9 kcal mol^−1^. The bluish (positive) regions appear both on the top and bottom portions of the O atom, which are lying perpendicular to the O_3_ plane, and are characterized by a *V_S,max_* of +20.9 kcal mol^−1^. In contrast, the two terminal O atoms in O_3_ accompany positive σ-holes that are occurring on the O–O bond extensions. These are weak: *V_S,max_* = +1.2 kcal mol^−1^ for each. The accompanying lateral portions of these atoms are less positive than the axial sites, with the *V_S,min_* associated with the two lone-pair regions on each terminal O atom being −8.3 and −12.3 kcal mol^−1^ (Figure S1e). These results clearly give insight into the amphoteric nature of the electron density distribution on the surfaces of the O atoms of the molecule.

The details of some of the selected maxima and minima of electrostatic potential on the van der Waals surfaces of some of the randomly chosen molecules (viz. OF_2_, (CN)OF, OBr_2_, CF_3_OF and O_3_) are given in the Supplementary Information (Figure S1).

**Figure 3:**
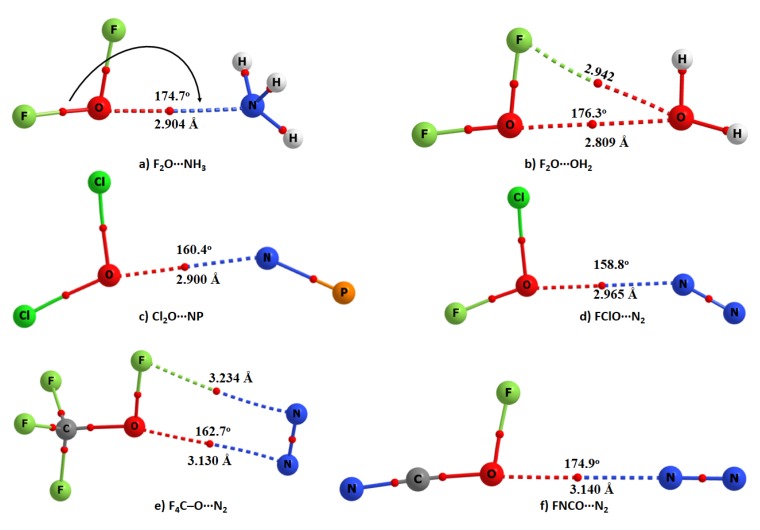
MP2/aug-cc-pVTZ molecular graphs of six binary complexes investigated. Bond paths and bond critical points are depicted as solid and dotted lines, and tiny red spheres, respectively. Selected intermolecular distances and the intermolecular angles of approach (∠N/O⋯O–X) (X = F, N, C) are shown.

Figure 3 illustrates six exemplar binary complexes that are driven not only by secondary interactions but also by O-centered chalcogen bonding. These complexes disprove the hypothesis that the oxygen atom in molecules does not form chalcogen bonding [34,35,36,37,38]. The following 13 characteristics clarify the presence of O-centered chalcogen bonding in all the six complexes examined, thus validating our statement.

### 3.1. Nature of the Intermolecular Distance

For the five N-linked binary complexes a and c–f shown in Figure 3, the N⋯O intermolecular distances were found ranging between 2.90–3.14 Å. For the F_2_O⋯OH_2_ complex shown in b), the O⋯O distance was 2.809 Å. Clearly, the N⋯O intermolecular distances in the N-linked complexes are all less than the sum of the van der Waals (vdW) radii of the O and N atomic basins, 3.16 Å (*r*_vdW_ (O) = 1.50 Å; *r*_vdW_ (N) = 1.66 Å [71]). Similarly, the O⋯O intermolecular distance in F_2_O⋯OH_2_ is less than twice the van der Waals radius of the O atom, 3.00 Å. Although the distance-based signature recommended for halogen bonding [30] is consistent with our result, it should be remembered that the error in the van der Waals radii of atoms is about 0.20 Å [33,44,45,72]. This means that the use of the criterion “less than the sum of the van der Waals criterion” for searching for a bonding interaction in a complex system may not always required to be satisfied for systems where reasonably weak interactions are play. This has been discussed in many occasions [33,44,45,72].

While not exact, the nature of the O⋯O interaction topology uncovered in F_2_O⋯OH_2_ could be analogous with that recently discussed by others [73]. The study has demonstrated that an interaction of this type plays an important role in the packing between molecular entities, leading to the formation of metal complexes in the solid state.

### 3.2. Directionality

The angle of approach for the formation of the N⋯O intermolecular interaction in five complexes of Figure 3 is lying between 158.8 and 174.9° (for FClO⋯N_2_ and NCFO⋯N_2_, respectively). For F_2_O⋯OH_2_, the angle of approach of the electrophile is such that ∠O⋯O–F = 176.3°. All of these contacts are typically Type-II [33], suggesting that directionality is also an important factor for the formation of an O-centered interaction. Type II noncovalent contacts (viz. Type II halogen bonds) are formed when the angle of attraction between the interacting donor and acceptor atomic basins varies largely between 160 and 180°, and the interacting atoms have opposite charge parity [33].

### 3.3. Nature of the Change in the Chalcogen Bond Donor Distance

The elongation of the chalcogen bond donor distance was observed for all of the dimers of Figure 3, except for FNCO⋯N_2_. For example, the F–O, Cl–O, and C–O covalent bond distances for the isolated monomers OF_2_, OCl_2_, and CF_3_OF were calculated to be 1.3989 Å, 1.7079 Å, and 1.3901 Å, respectively. The corresponding distances were 1.4109 Å, 1.7129 Å, and 1.3901 Å in the complexes F_2_O⋯NH_3_, Cl_2_O⋯N_2_, and CF_4_O⋯N_2_, respectively. These show a slight weakening of the chalcogen bond donor, accompanying the formation of the N⋯O chalogen bonds. By contrast, the formation of the NCFO⋯N_2_ complex is accompanied by a very marginal decrease in the C–O bond distance of 0.0001 Å (see Table 1 for Δ*r* values). Both the elongation and contraction of donor bond distance have been seen as a signature to validate the presence of a noncovalent interaction [74].

### 3.4. Nature of the Change in the Vibrational Frequency of the Chalcogen Bond Donor

**Figure 4:**
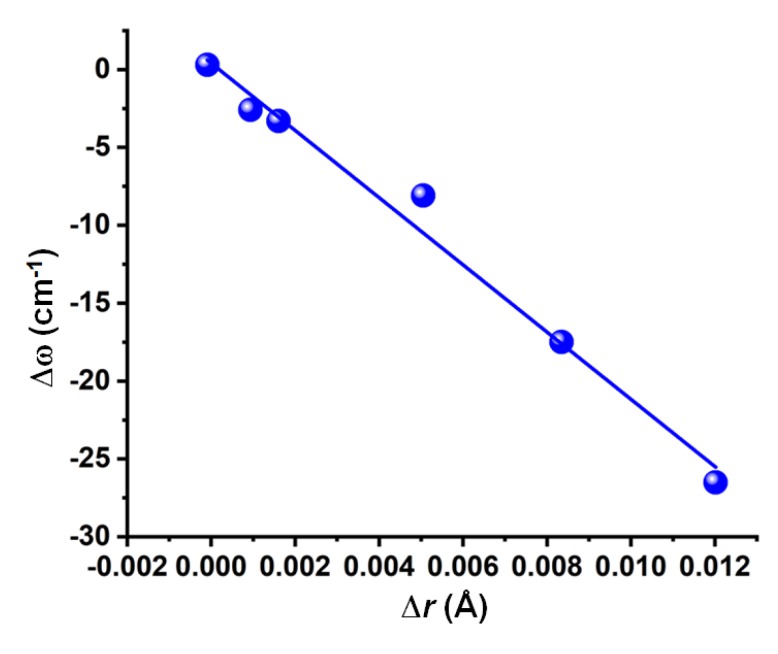
Dependence of the MP2/aug-cc-pVTZ level shift in the harmonic vibrational stretching frequency of the chalcogen donor bond (Δω) on the change in the chalcogen donor bond distance (Δ*r*) for the six O-bonded complexes of Figure 3. The Adj. R^2^ of the linear fit was 0.98.

Concomitant with the bond length elongation noted in Section 3.3, there was a vibrational red-shift in the F–O, Cl–O, and C–O bond stretching frequencies of the complexes a–e of Figure 3, except for NCFO⋯N_2_. The red-shift was the largest for F_2_O⋯NH_3_ (26.5 cm^−1^), and the smallest for CF_4_O⋯N_2_ (2.6 cm^−1^). The NCFO⋯N_2_ complex, on the other hand, displayed a vibrational blue-shift of 0.3 cm^−1^ in the C–O bond stretching frequency, which is consistent with the contraction of the corresponding bond noted in Section 3.3. Both the red-shift and blue-shift in the harmonic vibrational frequencies have been established as the signatures of noncovalent interactions [75]. The dependency of Δω on Δ*r* is shown in Figure 4.

For all the complexes, the intensity of the corresponding infrared band has increased (see ΔI values in Table 1).

### 3.5. The Molecular Electrostatic Surface Potential Signatures

The results of the MESP model in Figure 2 suggests that the formation of the complexes in Figure 3 is the result of attraction between sites of opposite electrostatic potential. The view is not surprising, given the positive σ-hole on O in each monomer faces the negative N/O sites in N_2_, NH_3_, PN, and OH_2_, causing the formation of the complexes. This is a typical signature routinely employed by Politzer et al [30,32,42,43,70,72] to rationalize the presence of σ-hole-centered noncovalent interactions in complex systems. Such a feature is prevalent regardless of the nature of the interaction, such as chalcogen bonding, pnictogen bonding, tetrel bonding, halogen bonding, or hydrogen bonding [32,33,41,44,45,70,72].

### 3.6. The QTAIM Signatures

The basic signatures [52] of QTAIM such as the presence of bonding pathways and bcp topology between the electrophilic O atom in the chalcogen bond donor molecules and the bases N/O atoms in the acceptor molecules are visible in all the molecular graphs shown in Figure 3. The charge density ρ_b_ at the N⋯O and O⋯O bcps was found to be very small, and the sign of both the Laplacian of the charge density (∇^2^ρ_b_) and the total energy density (H_b_) at the bcps was positive (see Table S1 of the Supplementary Information for details), indicating the closed-shell origin [76] of the N⋯O and O⋯O interactions. Note that the ∇^2^ρ_b_ is described as a *concavity detector* or as a *peak finder*—a positive value in the ∇^2^ρ indicates a minimum at the bcp, where the charge density is minimally concentrated.

### 3.7. Nature of Delocalization Index

QTAIM-based delocalization index (δ) analysis [77,78,79] gave very low values (0.0234–0.0519, Table S1) for the N⋯O and O⋯O atom–atom pairs in the complexes of Figure 3, and are typical for noncovalent interactions [77,78,79].

### 3.8. Nature of Reduced Density Gradient Isosurface Domains

**Figure 5:**
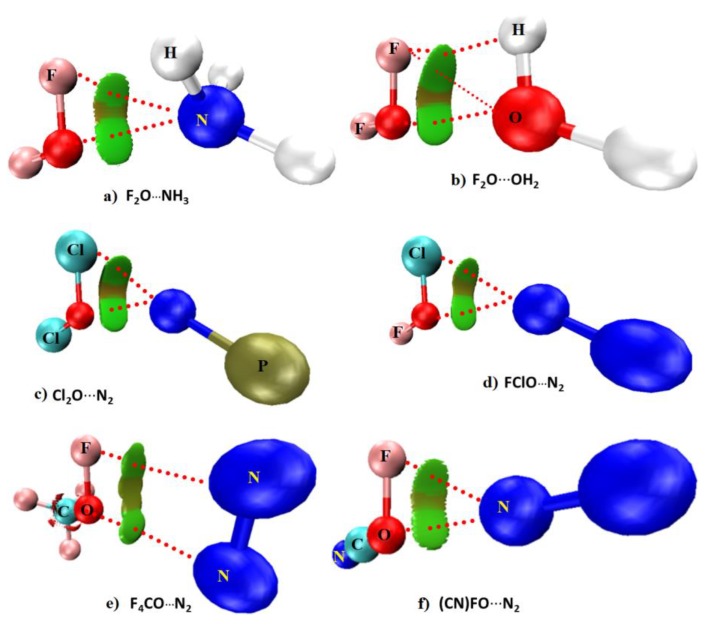
Illustration of the MP2/aug-cc-pVTZ level reduced density gradient (RDG) isosurface plots (0.5 au) for the six chalcogen-bonded complexes examined. The dotted lines in red represent possible intermolecular interactions, whereas the pseudo dumbbell-shaped volumes between the molecules represent the RDG domains. Color of RDG domains: green—weakly bound attractive attractions; brown—van der Waals. Atom labeling is shown.

The result of the RDG isosurface analysis [53] shown in Figure 5 confirms the presence of the chalcogen-bonding interactions between the O and N/O atoms of the six complexes. In fact, this analysis suggests the presence of both primary and secondary interactions between the monomers in each complex. The primary interactions are the genuine N⋯O and O⋯O chalcogen-bonding interactions in a, c–f, and b, respectively. These prevalent features are consistent with the common signatures of QTAIM (see Section 3.6 above). The greenish isosurfaces representing the primary interactions have the characteristic of sign(λ_2_)×ρ < 0, where λ_2_ is the second principal eigenvalue of the Hessian second-derivative charge density matrix.

As mentioned above, each complex comprises secondary interactions that follow a Type I or a Type III bonding topology [33]. Type I interactions are formed when interacting atomic basins generally have the same charge parity, and the angle of interaction varies between 90 and 150°. Type III interactions are formed when the interacting atoms are either both positive or both negative and the angle of interaction is similar to Type II (i.e., 160–180°). For example, the secondary interaction in the complex a is F⋯N type. This is a Type III contact, with ∠F⋯N–H = 170.4°. In b, the Type III contact predicted by QTAIM is O⋯F type, with ∠F⋯O–H = 168.3°. In c and d, the secondary interactions are both Cl⋯N type, and are Type III (∠Cl⋯N–P = 180.0° in c and ∠Cl⋯N–N = 178.7° in d). In e, an N⋯F Type I contact is observed as secondary, with ∠F⋯N–N = 95.1°. In f, it is F⋯N and is Type I (∠F⋯N–N = 152.3°). Although no QTAIM-based bonding pathway topology representing the secondary interactions is present in most complexes, the F⋯O and F⋯N secondary interactions are indeed apparent in the molecular graphs shown in b and e of Figure 3, respectively (see Table S2 of the Supplementary Information for QTAIM-based charge density properties and δ values). Needless to say, these interactions are the result of attraction between sites of unequal charge density delocalization, thus revealing that the anisotropy of charge density is an important aspect of their formation. The isosurfaces representing the secondary interactions are having the characteristics both of sign(λ_2_)×ρ < 0 and sign(λ_2_)×ρ > 0, which are described by the greenish and brownish volumes, respectively. These suggest that the results emerged from the MESP model alone (see Section 3.5 and Figure 2 above) are insufficient to explain the secondary interactions in all the six complexes examined, as it would only suggest the possibility of attraction to be occuring between sites of opposite electrostatic potential based on the argument that chalcogen bonding is a Coulombic interaction [27].

### 3.9. Nature of the Change in the Dipole Moment

The dipole moment μ was calculated to vary between 0.33 and 2.32 Debye for all the six complexes, indicating that they are all polar. From the changes in the dipole moment values Δμ shown in Table 1, it is clear that the formation of the binary complex is accompanied by either an increase or a decrease in the total dipole moment relative to the sum of the monomer dipole moments. For example, the increase in the dipole moment Δμ of F_2_O⋯NH_3_ relative to the sum of the total dipole moments of the isolated monomers F_2_O and NH_3_ was 0.003 Debye, whereas that of the CF_4_O⋯N_2_ complex relative to the sum of the dipole moments of the monomers of CF_4_O and N_2_ was 0.042 Debye. The remaining four complexes are accompanied by a decrease in the dipole moment compared to the sum of the monomer dipole moments, with the Δμ values lying between −0.001 and −0.878 Debye (for ClFO⋯N_2_ and Cl_2_O⋯NP, respectively). These features of polarity are typical of noncovalent interactions [80,81].

### 3.10. Effect of Polarizability on Complex Formation

Static polarizability α is an important property of molecular systems [82]. The data in Table 1 shows that the formation of the binary complex leads to an increase or a decrease in α, compared to the sum of the polarizabilities of the interacting monomers in each complex. The change Δα is found to be the lowest for CF_4_O⋯N_2_ (−0.11 au) and the largest for Cl_2_O⋯NP (1.62 au).

### 3.11. Nature of the Donor-Acceptor Natural Bond Orbital Interactions

The results of the second-order perturbative estimates of donor-acceptor (bond–antibond) charge transfer (CT) interactions in the NBO basis [55] suggested a weak charge transfer delocalization between the bonding orbitals associated with the Lewis base in N_2_, PN, NH_3_, and OH_2_, and the antibonding orbitals associated with the chalcogen bond donors. The second-order energies *E*^2^ associated with CT delocalizations were all less than 0.90 kcal mol^−1^. For instance, the CT delocalization between the NH_3_ and OF_2_ molecules is *n*(N) → σ*(O–F) (*E*^2^ = 0.65 kcal mol^−1^), and is responsible for the formation of the chalcogen bonding in the H_3_N⋯OF_2_ complex. The secondary interaction in this complex as revealed by RDG is described by *n*(F) → σ*(N–H), with an *E*^2^ of 0.24 kcal mol^−1^, where *n* refers the lone-pair orbital. The CT interactions responsible for the chalcogen bonding in the Cl_2_O⋯NP and ClFO⋯N_2_ complexes are *n*(N) → σ*(O–Cl) and π*(N–N) → σ*(O–C), respectively, with *E*^2^ values of 0.21 and 0.08 kcal mol^−1^, respectively. Similarly, the CT delocalizations are *n*(O) → σ*(O–F), *n*(O) → σ*(O–H), and *n*(F) → σ*(O–H) for the F_2_O⋯OH_2_ complex, with *E*^2^ values of 0.12 kcal mol^−1^, 0.22 kcal mol^−1^, and 0.16 kcal mol^−1^, respectively. One should not regard the CT interaction *n*(O1) → σ*(O4–H5) in this complex as a “back donation” (Text T3). This is expected given the O1 atom is neither entirely positive nor entirely negative and the CT delocalization is occurring between the lone-pair orbital on O1 and σ*(O4-H5) antibonding orbital in the partner molecule, thus representing the O⋯H hydrogen bond between them.

In any case, the CT interactions are *n*(N) → σ*(O–Cl), π(PN) → RY*(O), and *n*(N) → RY*(O) for PN⋯F_2_O, with *E*^2^ values of 0.21 kcal mol^−1^, 1.20 kcal mol^−1^, and 0.31 kcal mol^−1^, respectively. A detail of various other CT delocalizations explaining the secondary CT interactions in each binary complex is given in Text T3 of the Supplementary Information. Note that several complexes are accompanied with back donations from the O part of molecule in the acid fragment to the base part of the partner molecule, as observed, for example, for CF_4_O⋯N_2_ and ClFO⋯N_2_, which is not unexpected since the π*-orbital of N_2_ can accept electron density. Whereas the *E*^2^ values are small for weakly bound interactions, these are indeed larger than the threshold value of 0.05 kcal mol^−1^. Nevertheless, these results are also consistent with the RDG predicted isosurface topologies of intermolecular bonding interactions discussed above and thus cannot be overlooked.

### 3.12. Nature of the Complex Binding Energies

The uncorrected MP2 binding energies, Δ*E*(MP2), which were calculated using the supermolecular approach of Pople [83] vary between −1.86 and −0.81 kcal mol^−1^ for all of the complexes of Figure 3 (see Table 2 for values). The basis set superposition error energies, Δ*E*(MP2(BSSE)), accounted for by the counterpoise procedure of Boys and Bernardi [84] vary between −1.53 and −0.41 kcal mol^−1^ for the corresponding complexes, respectively. This result shows that the BSSE has some effect on the binding energy. Although this is not very marginal, the corrections for Δ*E*(MP2) are in the range of 0.32–0.48 kcal mol^−1^ for the six complexes. The BSSE on the uncorrected energy is found be maximal for the Cl_2_O⋯NP complex, with a BSSE value of 0.48 kcal mol^−1^.

From the Δ*E*(MP2(BSSE)) values in Table 2, it is obvious that the O-centered complexes are either weakly bound or van der Waals. However, the preference in the energy stability does not correlate with the strength of the positive σ-hole localized on the O atoms of the interacting monomers. This is understandable given that the *V_S,max_* values associated with the σ-hole on O in OF_2_, OCl_2_, OFCl, CF_3_OF and FOCN are +16.4 kcal mol^−1^, +3.5 kcal mol^−1^, +12.8 kcal mol^−1^, +2.4 kcal mol^−1^, and +11.3 kcal mol^−1^, respectively.

**Figure 6:**
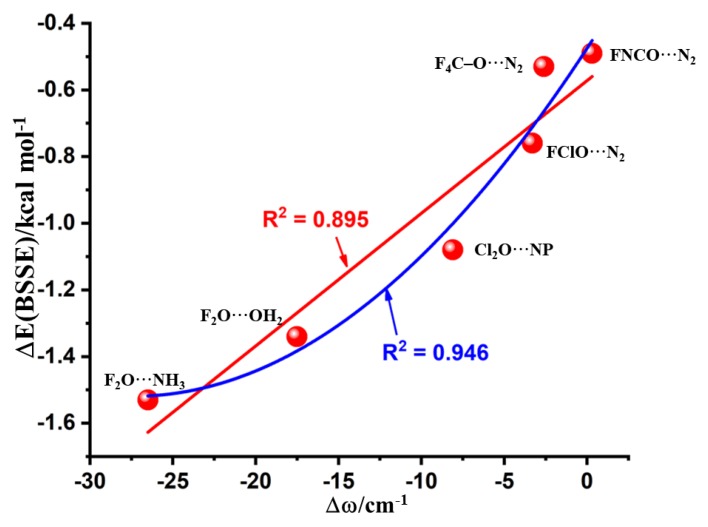
Dependence of the MP2/aug-cc-pVTZ level basis set superposition error energies (BSSE) corrected binding energy, Δ*E(BSSE)*, on the shift in the vibrational frequency associated with the chalcogen donor bond for all the six binary complexes. The red and blue lines represent the data fitted to the linear and quadratic equations, respectively.

Figure 6 shows the plot between Δ*E*(MP2(BSSE)) and Δω. The irregularity of the data points in the graph could be due to the secondary interactions that contribute to the interaction energies. Therefore, we have fitted the data to a linear equation, as well as that to a quadratic equation. The Adj. R^2^ values for the corresponding fits were 0.895 and 0.946, respectively, showing that the relationship between Δ*E*(MP2(BSSE)) and Δω could be better described by a quadratic function. A similar relationship between these properties was discussed elsewhere [85].

### 3.13. Factors Contributing to the Binding Energy of the Complexes: A SAPT Analysis

Previous suggestions show that halogen bonds are driven primarily by electrostatic forces [32,33,42,43,70]. A similar argument was provided for chalcogen bonds formed by the covalently bonded chalcogen atoms other than oxygen, in which case, the strength of the σ-hole is reasonably significant [34,35,36,37,38]. However, this view is not always true. This is indicative of the energy data of Table 2, and is not unexpected, given that the chalcogen bonds formed by the O atom of the molecules examined in this study conceive σ-holes that are not always strong. In any case, our DFT-SAPT-based EDA analysis [56] suggests that the interaction due to dispersion is the major driving force for the formation of the chalcogen-bonded complexes c–f of Figure 3, as |*E_eles_*| << |*E_disp_*|. For a and b, the role of electrostatics is more prominent; that is, *E_eles_* > *E_disp_*. This latter is not very surprising since the H atoms of the Lewis bases are also involved in the secondary engagements with the negative bases in OF_2_.

In contrast to the results above, the repulsive contribution, *E_rep_*, is seemingly very significant. This is true regardless of the nature of the complex systems being examined. Although the magnitude of this energy is found always to be larger than the *E_eles_*, the latter is certainly not just the contribution accounting for the stability of any of the six O-bonded complexes. For instance, the electrostatics, repulsion, induction, and dispersion contributions explaining the interaction energies of F_4_C–O⋯N_2_ (FNCO⋯N_2_) were −0.33 (−0.31), +1.05 (+0.71), −0.06 (−0.04), and −1.15 (−0.90) kcal mol^−1^, respectively, showing that the main driving force is dispersion. This means that chalcogen bonding cannot and should not always be regarded as purely electrostatically driven. In the same time, it should not be presumed that dispersion dominant interactions are not chalcogen bonds. Similar conclusions were previously made for halogen-bonding interactions formed by the most electronegative fluorine atom in molecules [41,44,45].

## 4. Conclusions

We conclude that the covalently bound oxygen in molecules does indeed feature an electron density-deficient region on its axial outer electrostatic surface, provided that the molecule contains some electron-withdrawing fragments. This electron density-deficient region is characterized by a positive or a negative electrostatic potential, but represents the valence-shell charge depletion in the Laplacian of the charge density distribution. Such a region refers to what one might call a σ-hole. We have shown that the “hole” of the bonded O atom of five randomly selected monomers has the ability to attract the Lewis bases of the interacting molecule, resulting in the formation of an O-centered chalcogen-bonding interaction. Moreover, the complex’s interaction energy is realized originating not only from the primary O-bonded interaction, but also from the contribution of the secondary interactions that play an important role in structural design. While we have presented 13 characteristics of the O-centered noncovalent interactions in this study, we are currently investigating to validate them in a series of 50 binary complexes that were formed between the O-centered monomers reported in this study and various Lewis bases containing the O, N, and X (X = F, Cl, Br) atoms. The study will be reported elsewhere.

Note that we recently investigated the possibility of chalcogen binding formed by the O atom in the OF_2_ molecule with 12 Lewis bases [86]. In this study, we discussed the characteristic features of chalcogen bonds based mainly on the geometric, energetic, and charge density properties. However, in the current study, we provided our perspective on a wide range of molecular scenarios, showing the nature and extent to which the local maximum of the electrostatic surface potential (associated with the s-hole) on the surface of the bound oxygen can be tuned. We also showed that the maxima of the electrostatic potential on the O atom responsible for the formation of chalcogen bonds in the complexes explored did not correlate with the binding energy and thus *V_S,max_* cannot be universally regarded as a measure of chalcogen bond strength as previously demonstrated for halogen-bonded systems. In addition, the results of the adopted NBO approach, along with the electronic (viz. dipole moment and polarizability) and vibrational property changes, which accompany the formation of chalcogen-bonded complexes provided an important insight into the origin of such interactions.

We further add that Scilabra et al. [28] have only recently reviewed the importance of the chalcogen bond in crystalline solids, wherein it was briefly speculated that the oxygen atom can nevertheless elicit electrophilicity and hence could form close contacts with nucleophiles. In particular, it was suggested that the crystal of (*S*,*S*)-(−)-2-methylsulfonyl-3-(2-chloro-5-nitrophenyl)oxaziridine [87] is driven by the Cl⋯O chalcogen bond and the crystal of Guanidinium 5-nitro(1,2,5)oxadiazolo(3,4-e)(2,1,3)benzoxadiazole-4-olate3,8-dioxide [88] is driven by the O⋯O chalcogen bond. While Scilabra et al. [28] have stressed the presence of O-centered chalcogen bonding in both the crystal systems, such a view might be misleading, since the anion moiety in the latter crystal is stabilized by the guanidinium cation.

## Figures and Tables

**Table 1:** Selected physical properties of the six binary complexes (Figure 3), obtained using MP2/aug-cc-pVTZ ^a,b^.

Complex	Bond	Distance (*r*)	Δ*r*	ω	I	Δω	ΔI	μ	α	Δμ	Δα
**F_2_O⋯NH_3_**	F–O	1.4109	0.0120	954.3	14.4	−26.50	1.64	1.82	28.18	0.003	0.53
**F_2_O⋯OH_2_**	F–O	1.4072	0.0084	963.3	12.0	−17.50	1.36	2.02	23.40	−0.150	0.31
**Cl_2_O⋯NP**	Cl–O	1.7129	0.0051	645.2	0.4	−8.10	0.27	2.32	67.08	−0.878	1.62
**FClO⋯N_2_**	F–O	1.4421	0.0016	858.8	21.5	−3.30	1.04	1.01	36.94	−0.001	0.35
**F_4_C–O⋯N_2_**	C–O	1.3911	0.0009	1240.1	358.6	−2.60	1.06	0.33	36.68	0.042	−0.11
**FNCO⋯N_2_**	C–O	1.3009	−0.0001	1039.5	1.3	0.30	1.6	1.69	37.98	−0.175	0.48

^a^ The properties include the distance of the chalogen bond donor in the complex (*r*/Å), the harmonic vibrational frequency (ω/cm^−1^), the infrared band intensity (I/km mol^−1^), the complex dipole moment (μ/Debye), and the complex polarizability (α/au). The changes in these complex properties with respect to that found in the isolated monomer molecules are given as Δ*r*/Å, Δω/cm^−1^, ΔI/ km mol^−1^, Δμ/Debye and Δα/au, respectively. ^b^ Δ*X* (*r*, ω) = *X*_complex_ − *X*_monomer_; Δ*X* (*μ*, α) = *X*_complex_ − Σ*X*_monomers_; ΔI = I_complex_/I_monomer_.

**Table 2:** Comparison of the DFT-SAPT decomposed energy components and the total SAPT0 interaction energies with the MP2 computed uncorrected and corrected binding energies for the six binary complexes ^a^.

Complex	Figure 2	*E_eles_*	*E_rep_*	*E_pol_*	*E_disp_*	Δ*E*(SAPT0)	Δ*E*(MP2)	Δ*E*(MP2(BSSE))
F_2_O⋯NH_3_	a	−2.65	3.14	−0.65	−1.61	−1.77	−1.86	−1.53
F_2_O⋯OH_2_	b	−1.81	1.87	−0.37	−1.35	−1.66	−1.66	−1.34
Cl_2_O⋯NP	c	−0.69	3.02	−0.57	−2.66	−0.91	−1.56	−1.08
FClO⋯N_2_	d	−0.64	1.40	−0.14	−1.42	−0.80	−1.12	−0.76
F_4_C–O⋯N_2_	e	−0.34	1.05	−0.08	−1.22	−0.59	−0.88	−0.53
FNCO⋯N_2_	f	−0.31	0.71	−0.04	−0.90	−0.54	−0.81	−0.49

^a^ Values in kcal mol^−1^.

## Supplementary Materials

The following are available online at https://www.mdpi.com/1420-3049/24/17/3166/s1, Figure S1: The MP2/aug-cc-pVTZ level 0.001 a.u. isodensity envelope mapped potential extrema on the surface of some selected monomers; Table S1: Selected MP2/aug-cc-pVTZ QTAIM properties of the O⋯N/O⋯O bonded interactions in the six O-bonded complexes of Figure 3, including the charge density (ρ_b_), the Laplacian of the charge density (∇^2^ρ_b_), the total energy density (H_b_) and the delocalization index (δ). Table S2: Selected MP2/aug-cc-pVTZ QTAIM properties of the secondary interactions in the six O-bonded complexes of Figure 3, including the charge density (ρ_b_), the Laplacian of the charge density (∇^2^ρ_b_), the total energy density (H_b_) and the delocalization index (δ); Text T1: MP2/aug-cc-pVTZ optimized redundant internal coordinates of all the 21 monomers examined; Text T2: MP2/aug-cc-pVTZ optimized redundant internal coordinates of all the six binary complexes analyzed; Text T3: RHF/aug-cc-pVTZ level results of the second-order perturbative estimates of donor-acceptor (bond-antibond) interaction energies, *E*^2^ (values in kcal mol^−1^), in the NBO basis.
