# Supplementary material for: Does Oxygen Feature Chalcogen Bonding?
Source: Molecules. 2019 Aug 30;24(17):3166. doi: 10.3390/molecules24173166 (PMC6749412; doi:10.3390/molecules24173166)
Supplement: Supplementary file 1 [file molecules-24-03166-s001.pdf]

molecules

Does Oxygen Feature Chalcogen Bonding?

Pradeep R. Varadwaj [1,2,*]

[1] Department of Chemical System Engineering, School of Engineering, The University of Tokyo 7-3-1, Tokyo 113-8656, Japan; prv.aist@gmail.com or pradeep@t.okayama-u.ac.jp
[2] The National Institute of Advanced Industrial Science and Technology (AIST), Tsukuba 305-8560, Japan

Supplementary Information

Text T1: MP2/aug-cc-pVTZ optimized redundant internal coordinates of all the 21 monomers examined (see Figure 1 of main article for molecule type). The charge and multiplicity of each complex are 0 and 1, respectively.

a) Fluorine nitrate

N,0,-0.4307186979,-0.4264829318,-0.0913176985
O,0,-0.0387782041,-1.5468130111,-0.1851404783
O,0,-0.7147249571,0.4520474957,-0.8324608216
O,0,-0.5839356883,-0.1112654373,1.417083038
F,0,-1.0492158329,1.2191049453,1.4776613495

b) Perchloryl fluoride

Cl,0,-0.931056139,0.2867195219,-0.1577186297
F,0,-0.378227969,1.0685160401,1.1964041566
O,0,-0.3720338407,-1.008796893,0.0072241377
O,0,-2.3388191315,0.3819665877,0.0072243344
O,0,-0.3720083714,1.0773119885,-1.19719156

c) Tetrafluoroacetic acid

C,0,-2.100808421,-1.6290549648,0.0445747798
C,0,-1.6606053767,-0.6176392957,1.1317660195
O,0,-1.8630842318,0.557167775,1.0788189955
O,0,-0.9935541267,-1.0964585791,2.2303349963
F,0,-0.8410668652,-2.512394584,2.125560187
F,0,-2.9433231261,-2.5318853074,0.5438315013
F,0,-2.7201797459,-0.9565710285,-0.9137625896
F,0,-1.0483663166,-2.2513669255,-0.4842296199

d) Bis(fluorooxy)difluoromethane

C,0,-1.6762195052,-0.4982642433,-0.0450548402
F,0,-2.9572835322,-0.8215183309,0.0423171952
F,0,-0.9386956296,-1.5901319435,0.0291890978
O,0,-1.473162084,0.3707746508,1.0210703216
O,0,-1.3965724779,0.2393100166,-1.185001631
F,0,-1.5586151724,-0.6872711097,-2.2561920971
F,0,-0.0686698386,0.5983562499,1.0439738837

e) Trifluoromethyl hypofluorite

C,0,-2.6038639136,-0.3705147953,-0.0024991957
F,0,-3.9253305374,-0.3651072844,-0.0101538729
F,0,-2.1721350303,0.2538318222,-1.084251596
F,0,-2.1514527173,-1.6113120549,0.0209406058
O,0,-2.086475326,0.1849164163,1.1620654548
F,0,-2.5685885055,1.5231722061,1.146224764

f) Difluorochloromethyl hypofluorite

O,0,0.457573,-1.104741,0.
F,0,1.888574,-1.010255,0.
C,0,0.029259,0.213985,0.
F,0,0.457573,0.869693,1.073172
F,0,0.457573,0.869693,-1.073172
Cl,0,-1.709978,0.058344,0.

g) 1,1-Bis(fluorooxy)tetrafluoroethane

C,0,-0.1295927098,0.0333471399,0.1070115216
O,0,0.1518330341,-1.2545130074,0.5383279247
O,0,0.7626526975,1.0186725277,0.5033454347
F,0,-1.3881970084,0.3759435127,0.3946631261
F,0,0.5189228342,1.2464457127,1.8897620243
F,0,-0.1588191407,-1.2882483718,1.9296694634
C,0,0.0603330525,-0.0415667614,-1.4418146593
F,0,-0.2173033775,1.1429907927,-1.9690127848
F,0,-0.7753786311,-0.9443191327,-1.9366613341
F,0,1.3066987593,-0.3795140424,-1.7473173565

h) Fluoro(fluorooxy)oxomethane

C,0,-1.4552008494,0.4688632478,0.0327467258
O,0,-0.2728002149,0.4453653494,0.0279706489
O,0,-2.1611417497,-0.6840525873,-0.1506822722
F,0,-2.222584397,1.5199117369,0.2008231165
F,0,-3.5599006489,-0.4210218367,-0.108454069

i) Trifluoromethyl peroxynitrate

O,0,-0.7591636404,-0.0403027132,-0.9507434143
O,0,0.2456440747,0.8435994609,-0.5069974932
C,0,1.2840488407,0.0612294683,-0.0251921393
N,0,-1.869155476,-0.0854702576,0.1965163471
O,0,-1.6486376931,0.602897033,1.1325260595
O,0,-2.7280049341,-0.8235922533,-0.1607213724
F,0,1.8095697118,-0.7008340618,-0.972622591

F,0,2.1880136259,0.9191732306,0.4112036564
F,0,0.8941674404,-0.727506077,0.9711251171

j) Bis(trifluoromethyl) trioxide
O,0,-0.9945781339,0.0634542567,0.0022143858
O,0,0.4477048464,0.2158237226,-0.0002588544
O,0,-1.3323936909,1.4738737033,0.0020227921
C,0,-2.7166680549,1.4876524312,-0.0005135273
C,0,0.922775574,-1.0844529981,-0.0001991082
F,0,-3.0328367885,2.7682883886,-0.0001200958
F,0,-3.2222948179,0.8943226674,1.0721581947
F,0,-3.2184154084,0.895957045,-1.0758938286
F,0,0.5341523639,-1.756345236,1.0749019961
F,0,0.5306171308,-1.7577262133,-1.0731507826
F,0,2.2354934198,-0.9549998877,-0.0024406718

k) Fluorooxy hypofluorite
O,0,-0.4711664449,-0.5671794293,0.
O,0,0.4711664449,0.5671794293,0.
F,0,1.6675342061,-0.1678619936,0.
F,0,-1.6675342061,0.1678619936,0.

l) Cyanic cyanate
O,0,-0.4304980562,0.8029537088,0.
C,0,0.8872450178,0.7355436165,0.
C,0,-0.933913286,2.0226110525,0.
N,0,2.0503681894,0.5897658246,0.
N,0,-1.4595879767,3.0703575851,0.

m) Cyano hypofluorite
O,0,-0.6419191169,0.7261850511,0.
C,0,-0.9689040397,1.9853999045,0.
N,0,-1.3472154818,3.0983984451,0.
F,0,0.8161636385,0.7223595093,0.

n) Cyano hypochlorite
O,0,-0.5573010398,0.7602673227,0.
C,0,-0.9521699791,2.0030087851,0.
N,0,-1.3871842598,3.095145931,0.
Cl,0,1.1647802787,0.6739208712,0.

o) Cyano hypobromite

O,0,-0.5425308763,0.769745685,0.

C,0,-0.9499462703,2.0051947438,0.

N,0,-1.3968616223,3.0932214882,0.

Br,0,1.3074637689,0.664180993,0.

p) Difluorine monoxide

O,0,0.,0.,0.60314

F,0,0.,1.094471,-0.268062

F,0,0.,-1.094471,-0.268062

q) Fluorochloro monoxide

O,0,0.,-0.083500464,-0.7607312388

Cl,0,0.,1.2870621557,0.2076178127

F,0,0.,-1.2035616917,0.144989336

r) Fluorobromo monoxide

O,0,0.,-0.1296628434,-0.7840673683

Br,0,0.,1.367952606,0.2398516131

F,0,0.,-1.2382897625,0.1360916653

s) Dichlorine monoxide

O,0,0.,0.,-0.7875059385

Cl,0,0.,1.4006530636,0.1896909243

Cl,0,0.,-1.4006530636,0.1896909243

t) Dibromine monoxide

O,0,0.,0.,-0.8243116243

Br,0,0.,1.5228265928,0.2080937671

Br,0,0.,-1.5228265928,0.2080937671

u) Ozone

O,0,1.092713,-0.224768,0.

O,0,0.,0.449243,0.

O,0,-1.092713,-0.224476,0.

Text T2: MP2/aug-cc-pVTZ optimized redundant internal coordinates of all the six binary complexes analyzed (see Figure 3 of main article). The charge and multiplicity for each complex are 0 and 1, respectively.

a) $F_2O \cdots NH_3$

O,0,0.6595290156,-0.215709879,0.

F,0,2.0692446009,-0.1570837364,0.

F,0,0.3020159124,1.1457867281,0.

N,0,-2.2190324714,-0.6026059432,0.

H,0,-2.9428682271,-1.3104599265,0.

H,0,-2.3815153028,-0.019492831,-0.8117532178

H,0,-2.3815153028,-0.019492831,0.8117532178

b) $F_2O \cdots OH_2$

O,0,0.0418857676,-0.6233687054,0.0340653803

F,0,-0.6997313382,-1.8117470759,0.1684209116

F,0,-0.9505013133,0.3180882469,-0.2963760015

O,0,1.6712109194,1.651673692,-0.2140871077

H,0,2.4253880464,2.240139539,-0.1176749272

H,0,0.9298379182,2.2396773034,-0.3861012555

c) $Cl_2O \cdots NP$

O,0,-1.4380909241,-0.2093902234,0.0034688143

Cl,0,-2.9801092513,-0.9551944926,-0.0019706433

Cl,0,-1.6157805173,1.4890239745,-0.0002785936

N,0,1.4445367126,0.1034066002,0.005357759

P,0,2.8387139801,-0.5278328587,0.0079266636

d) $FClO \cdots N_2$

O,0,-0.848421,-0.217687,0.00072

F,0,-2.152217,-0.833813,-0.000688

Cl,0,-1.128755,1.437776,0.00027

N,0,2.109101,-0.007475,0.000296

N,0,3.116395,-0.483946,-0.000568

e) F_4C–O···N_2

O,0,0.03994,0.180616,0.

F,0,-1.380526,0.096565,0.

C,0,0.454864,-1.147114,0.

F,0,0.03994,-1.795394,1.07374

F,0,0.03994,-1.795394,-1.07374

F,0,1.772411,-1.056416,0.

N,0,0.036113,3.311069,0.

N,0,-1.078197,3.316576,0.

f) FNCO···N_2

N,0,-0.9579035756,1.8874852603,-0.1586617942

N,0,-1.9467988951,2.3811464771,-0.2999283669

O,0,1.823740076,0.447207891,-0.000543347

F,0,0.9496364716,-0.7184145755,-0.0581643875

C,0,3.0263004838,-0.0478062926,0.0314892128

N,0,4.1432495793,-0.4134622503,0.0632425029

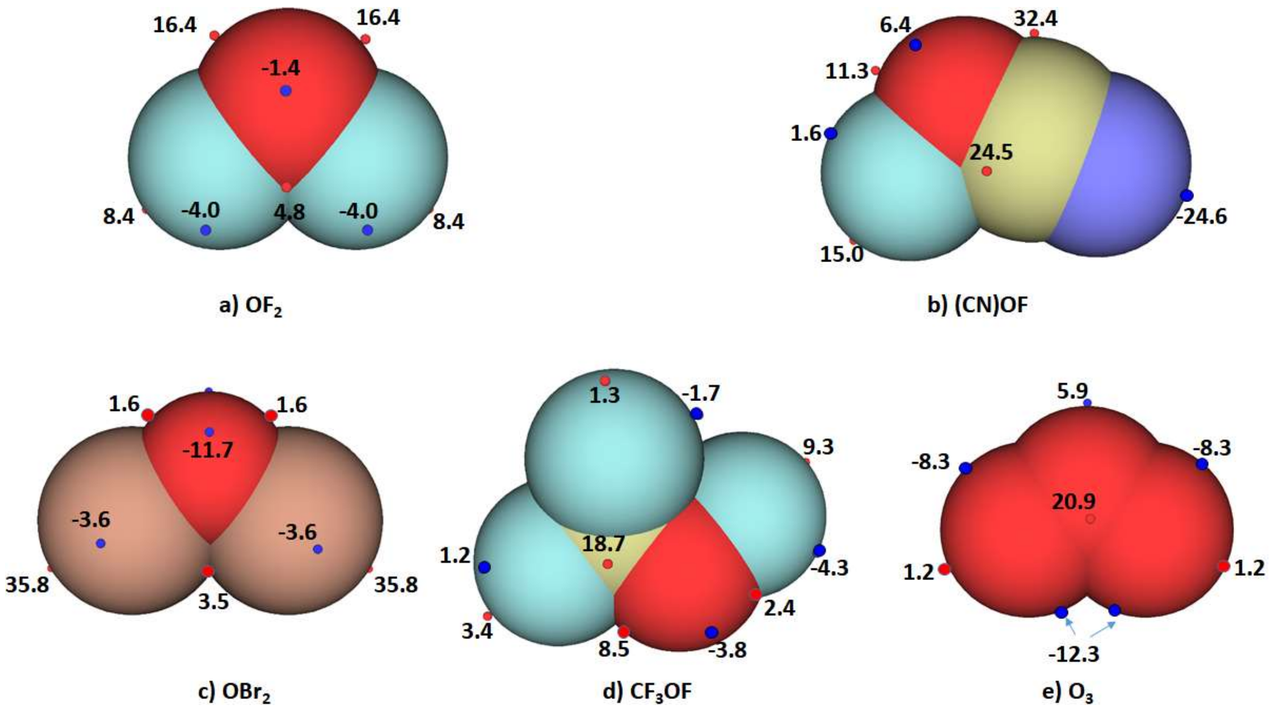

Figure S1. The MP2/aug-cc-pVTZ level 0.001 a.u. isodensity envelope mapped potential extrema on the surface of some selected monomers. The tiny blue and red circles represent $V_{S,min}$ and $V_{S,max}$, respectively. Atom color: C – light-yellow; O – red: N – blue; F – Cyan.

Table S1. Selected MP2/aug-cc-pVTZ QTAIM properties of the O···N/O···O bonded interactions in the six O-bonded complexes of Figure 3, including the charge density (ρ_b), the Laplacian of the charge density ($\nabla^2\rho_b$), the total energy density (H_b) and the delocalization index (δ).

Complex	Figure 3	ρ_b/au	$\nabla^2\rho_b$/au	H_b/au	δ
$F_2O\cdots NH_3$	a	0.0085	0.0381	0.0022	0.0519
$F_2O\cdots OH_2$	b	0.0073	0.0408	0.0024	0.0381
$Cl_2O\cdots NP$	c	0.0084	0.0384	0.0022	0.0488
$FClO\cdots N_2$	d	0.0062	0.0307	0.002	0.0347
$F_4C–O\cdots N_2$	e	0.0046	0.0207	0.0013	0.0235
$FNCO\cdots N_2$	f	0.0044	0.0213	0.0014	0.0234

Table S2. Selected MP2/aug-cc-pVTZ QTAIM properties of the secondary interactions in the six O-bonded complexes of Figure 3, including the charge density (ρ_b), the Laplacian of the charge density ($\nabla^2\rho_b$), the total energy density (H_b) and the delocalization index (δ).[a.]

Complex	Figure 2	ρ_b/au	$\nabla^2\rho_b$/au	H_b/au	δ
$F_2O\cdots NH_3$	F3···N4				0.0270
$F_2O\cdots OH_2$	F3···O4	0.0065	0.0329	0.0016	0.0289
	F3···H6				0.0045
$Cl_2O\cdots NP$	Cl3···N4				0.0488
$FClO\cdots N_2$	Cl3···N4				0.0240
$F_4C–O\cdots N_2$		0.0036	0.0156	0.0008	0.0168
$FNCO\cdots N_2$	N1···F4				0.0175

[a] Atom labeling is shown in the ball-and-stick models of Text T3.

Text T3: RHF/aug-cc-pVTZ level results of the second-order perturbative estimates of donor-acceptor (bond-antibond) interaction energies, E^2 (values in kcal mol^{-1}), in the NBO basis. The symbols LP and BD refer to the lone-pair and bonding orbitals, respectively. Similarly, BD* and RY* refer to anti-bonding and Rydberg-type orbitals, respectively. Values in the parentheses represent the involvement of the specific lone-pair/anti-bonding orbital. For example, LP(1) and LP(3) represent the lone-pair σ and π orbitals, respectively, whereas BD*(1) and BD*(2) represent the σ* and π* anti-bonding orbitals, respectively. Atom-type and -labeling is shown for each complex.

a) **$F_2O\cdots NH_3$**

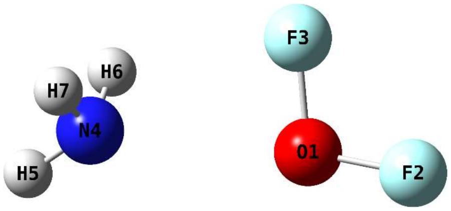

Donor		Acceptor	E^2
LP (1) N4	→	BD*(1) O 1 - F 2	0.65
LP (3) F3	→	BD*(1) N 4 - H 5	0.24
LP (1) O1	→	BD*(1) N 4 - H 5	0.15

b) **$F_2O\cdots OH_2$**

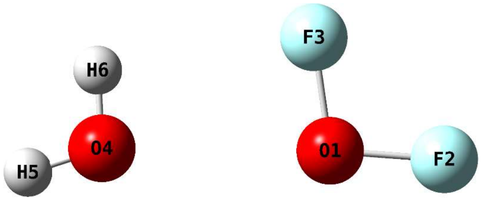

Donor		Acceptor	E^2
LP (1) O4	→	BD*(1) O1 - F2	0.12
LP (2) O4	→	BD*(1) O1 - F2	0.06
LP (1) O1	→	BD*(1) O4 - H5	0.22
LP (3) F3	→	BD*(1) O4 - H5	0.16

c) **$Cl_2O\cdots NP$**

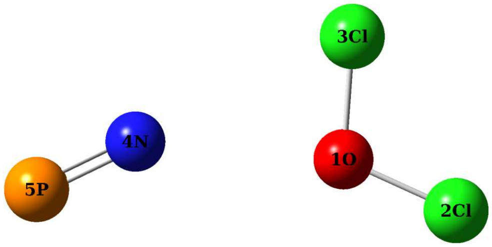

Donor		Acceptor	E^2
LP (1) N4	→	BD*(1) O1-Cl2	0.21
BD (3) N4 - P5	→	RY*(1) O1	0.85
BD (3) N4 - P5	→	RY*(3) O1	0.35
LP (1) N4	→	RY*(1) O1	0.19
LP (1) N4	→	RY*(3) O1	0.12

d) **$FClO\cdots N_2$**

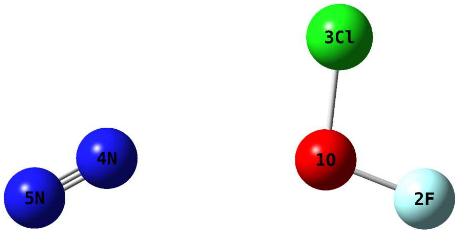

Donor		Acceptor	E^2
BD (3) N4 - N5	→	RY*(5) O1	0.25
LP (1) N4	→	BD*(1) O1 - F2	0.09
LP (1) N4	→	BD*(1) O1 -Cl3	0.05
BD (1) O1 - Cl3	→	RY*(2) N4	0.67
BD (1) O1 - Cl3	→	RY*(1) N4	0.21
BD (1) O1 - F2	→	RY*(4) N4	0.11

e) **$F_4C{-}O\cdots N_2$**

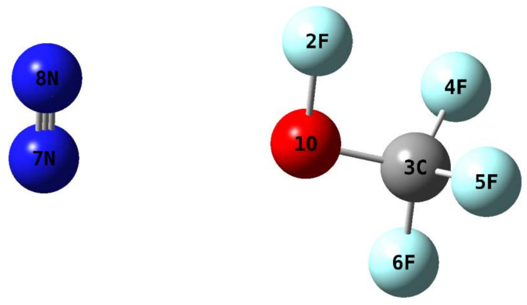

Donor	Acceptor	E^2
BD (3) N7 - N8 →	BD*(1) O1 - C3	0.08
BD (3) N7 - N8 →	RY*(6) O1	0.07
BD (1) O1 - F2 →	RY*(2) N7	0.05
BD (1) O1 - F2 →	RY*(2) N8	0.10
LP (2) O1 →	RY*(3) N7	0.14
LP (1) O1 →	BD*(2) N7 - N8	0.08
LP (3) F2 →	BD*(2) N7 - N8	0.10
LP (1) O1 →	BD*(2) N7 - N8	0.08

f) **$FNCO\cdots N_2$**

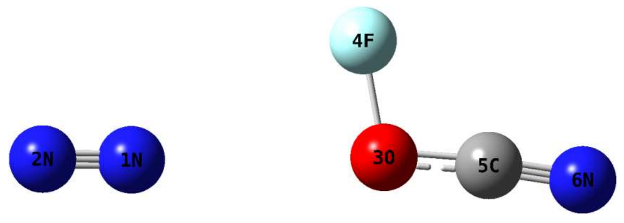

Donor	Acceptor	E^2
BD (3) N1 - N2 →	RY*(4) O3	0.06
BD (3) N1 - N2 →	RY*(6) O3	0.16
LP (1) N1 →	RY*(13) O3	0.05
LP (1) N1 →	RY*(6) O3	0.06
LP (1) O3 →	BD*(3) N1 - N2	0.08
BD (1) O3 - C5 →	RY*(3) N1	0.13
BD (1) O3 - C5 →	RY*(1) N1	0.07
